# p19Arf sensitizes B16 melanoma cells to interferon-β delivered via mesenchymal stem cells *in vitro*

**DOI:** 10.1590/1414-431X20198876

**Published:** 2020-02-14

**Authors:** R.C. Da-Costa, I.L. Vieira, A. Hunger, R.E. Tamura, B.E. Strauss

**Affiliations:** 1 Viral Vector Laboratory, Centro de Investigação Translacional em Oncologia/LIM24, Instituto do Câncer do Estado de São Paulo, Faculdade de Medicina, Universidade de São Paulo, São Paulo, SP, Brasil; 2 Biotecnologia Unidade 1, Cristália Produtos Químicos Farmacêuticos, Itapira, SP, Brasil; 3 Departamento de Ciências Biológicas, Universidade Federal de São Paulo, Diadema, SP, Brasil

**Keywords:** Gene therapy, Immunotherapy, Stem cell therapy, Adenovirus, p53, Interferon-β

## Abstract

The immune stimulatory and anti-neoplastic functions of type I interferon have long been applied for the treatment of melanoma. However, the systemic application of high levels of this recombinant protein is often met with toxicity. An approach that provides localized, yet transient, production of type I interferon may overcome this limitation. We propose that the use of mesenchymal stem cells (MSCs) as delivery vehicles for the production of interferon-β (IFNβ) may be beneficial when applied together with our cancer gene therapy approach. In our previous studies, we have shown that adenovirus-mediated gene therapy with IFNβ was especially effective in combination with p19Arf gene transfer, resulting in immunogenic cell death. Here we showed that MSCs derived from mouse adipose tissue were susceptible to transduction with adenovirus, expressed the transgene reliably, and yet were not especially sensitive to IFNβ production. MSCs used to produce IFNβ inhibited B16 mouse melanoma cells in a co-culture assay. Moreover, the presence of p19Arf in the B16 cells sensitizes them to the IFNβ produced by the MSCs. These data represent a critical demonstration of the use of MSCs as carriers of adenovirus encoding IFNβ and applied as an anti-cancer strategy in combination with p19Arf gene therapy.

## Introduction

Cancer therapy using recombinant cytokine proteins has been shown to mediate antineoplastic effects, but may present toxicity when high level systemic administration is used. In the case of type I interferon, the recombinant protein is expected to promote innate and adaptive anti-tumor immune responses, induce apoptosis in cancer cells, and inhibit angiogenesis (1). Interferon (IFN)α therapy was approved by the US Food and Drug Administration (FDA) in 1995, and it has been shown to reduce melanoma tumor burden and prolong survival in some patients (2). The short half-life of IFNα limits therapeutic activity and, thus, high levels of recombinant protein are required. However, a high systemic dosage of IFNα is associated with adverse effects including myalgia, pyrexia, and depression (3).

Alternatively, a variety of gene transfer methods may be employed in order to achieve high local concentration of type I interferon while avoiding systemic toxicity. Such approaches include the intratumoral application of non-replicating viral vectors, use of armed oncolytic viruses or even mesenchymal stem cells (MSCs) as carriers of vectors encoding type I interferon (1). For the latter, autologous MSCs may be isolated from the circulation, bone marrow, or adipose tissue, including lipoaspirates, cultivated in the laboratory, modified and expanded *ex vivo*, and then infused in the patient (4–6). The use of MSCs as a delivery vehicle for the therapeutic payload has been shown to home to and infiltrate the tumor site where they provide therapeutic transgene levels while shielding the gene transfer vector from immune recognition (7–11).

In our previous studies, we have shown that direct intratumoral delivery of an adenoviral vector encoding IFNβ inhibits tumor progression (12,13). Moreover, the association of IFNβ gene transfer along with p19Arf (alternative reading frame of the *Cdkn2a* gene, p19Arf in mice, p14ARF in humans) brought the added benefit of immunogenic cell death and the combined, but not individual, treatments served as an immunotherapy (12–14). The role of p19Arf is to block the interaction of Mdm2 with p53, sparing p53 from ubiquitination and subsequent degradation, thus promoting its activation and tumor suppressor function (15). The type I interferon and p53/p19Arf pathways are known to cooperate in the induction of anti-viral defense and cell death (16,17). In addition, the adenoviral vectors used, AdRGD-PG, were modified to contain improvements at both the transductional (RGD-modified fiber protein) and transcriptional levels (p53-responsive chimeric promoter, PG) (13). In this way, the vector and transgenes are expected to work together to promote high level expression, cell death, and anti-tumor immune modulation, points that have been substantiated previously (18,19).

Here, we explored a novel approach to the use of IFNβ plus p19Arf gene transfer. MSCs derived from mouse adipocyte tissue were transduced with AdRGD-PG-IFNβ and used to deliver this secreted, immune modulatory protein to mouse melanoma cells *in vitro*. We showed that MSCs tolerate IFNβ production relatively well, while B16 melanoma cells were inhibited in the presence of the IFNβ produced by the MSCs. Moreover, B16 cells previously treated with the AdRGD-PG-p19Arf vector were further sensitized to the effects of IFNβ produced by MSCs. These assays suggested that MSCs may be an interesting vehicle for the delivery of IFNβ to tumor cells, especially in combination with p19Arf gene transfer.

## Material and Methods

### Cell culture

The B16F10 mouse melanoma cells (referred to simply as B16) were kindly provided by Roger Chammas (ICESP, FM-USP) and verified as per Hunger et al. (13). The B16 cells were cultivated in RPMI containing a 1× concentration of the commercial product Antibiotic-Antimycotic and 10% fetal calf serum (FBS) (all products from Thermo Fisher, USA). To generate the B16-GFP cell line, B16 cells were transduced with Lego-G/puro-opt (kindly provided by Kristoffer Reicken, University Medical Center, Germany) at a MOI of 1 and then selected for puromycin resistance as per Vieira et al. (20).

### Isolation and cultivation of MSC

All animal procedures were evaluated and approved by the Commission for the Ethical Use of Animals (CEUA) of FM-USP, protocol 177/14. Animals were supplied by the Biotério Central (FM-USP) and housed at the Biotério Experimental, Centro de Medicina Nuclear (CMN, FM-USP). The animals used were 6-week-old male C57BL/6 mice, that were allowed access to food and water *ad libitum*.

For MSC isolation, a single animal was euthanized by CO_2_ inhalation and immediately subcutaneous adipose tissue (AT) was collected and washed with 1× phosphate buffered saline (PBS) containing 2.5× antibiotic-antimycotic (Thermo Fisher). Then, the AT was incubated in the presence of 0.075% collagenase 1 (Sigma-Aldrich, USA) diluted in 1× PBS for 30 min, at 37°C with agitation before adding Dulbecco's modified Eagle medium - Nutrient Mixture F-12 (DMEM/F-12) containing 20% FBS (Thermo Fisher). The treated AT was then pelleted (300 *g*, 10 min) before resuspension in fresh medium (DMEM/F12, 20% FBS) and transferred to culture dishes. At 48 h intervals, the supernatant and non-adherent cells were discarded and the adherent cell population was washed with 1×PBS and fresh medium was added, until 80% confluence was reached. Cells were then trypsinized using TrypLE (Thermo Fisher). This yielded approximately 4×10^6^ MSC at passage 3. We observed that at each passage, the cells had doubled 2 to 3 times. This permitted the cryopreservation of 6 vials, each with approximately 1×10^6^ cells at passage 4. These could then be thawed and expanded as needed for the assays performed at passage 5 or 6.

### Cell surface markers

MSCs, 1×10^5^/1.5 mL tube, were stained with the antibodies indicated in Supplementary Table S1 using a 1:100 dilution in 1×PBS containing 1% bovine serum albumin (BSA) at 4°C for 30 min in the dark. Then, the cells were washed three times with 1× PBS before flow cytometry (Attune, Thermo Fisher) and analyzed using the Attune Cytometric Software.

### Differentiation

The MSCs were plated, 1×10^4^ cells/well in 6-well dishes, and cultivated until reaching 50% confluence. Then, the specific differentiation medium (StemPRO, Thermo Fisher) was added and the cells were incubated before staining to reveal the cell type. For adipogenesis, incubation was carried out at 37°C for 14 days before fixation with paraformaldehyde and staining with Oil Red O (Sigma-Aldrich). For chondrogenesis, after 14 days of incubation at 37°C, the cells were treated with toluidine blue (Sigma-Aldrich) before washing and successive fixation steps using 70%, 90%, and absolute ethanol. In the case of osteogenesis, the cells were incubated at 37°C for 21 days before adding 1% silver nitrate and treating with UV light for 45 min, washing, treating with 3% sodium thiosulfate, and finally adding Van Gieson stain (Merck, Germany), and then washing with absolute ethanol.

### Vectors, production, and titration

The AdRGD-CMV-LacZ vector was kindly provided by Dr. Hiroyuki Mizuguchi (Osaka University, Japan) (21). The AdRGD-PG-eGFP, AdRGD-PG-p19, and AdRGD-PG-IFNβ vectors encoding enhanced green fluorescent protein (GFP), mouse p19Arf, or mouse IFNβ, respectively, have been described previously (12–14). Virus production was performed using iodixanol gradient centrifugation (209,627 *g*, 1 hour, 10°C) (22) and titration was performed using the AdenoX Rapid Titer kit (Clontech, USA). The biological titer, reported as infectious virus particles (IVP)/mL, was used to calculate the multiplicity of infection (MOI).

### Transduction

When working in 6-well dishes, 5 ×10^4^ cells/well were plated and allowed to incubate at 37°C for 24 h. Then, the medium was removed and replaced with fresh medium containing 2% FBS plus the virus stock, using a total volume of 600 µL. After 4 h incubation at 37°C, the medium was removed and replaced with 2 mL fresh DMEM/F12, 20% FBS.

To control for total MOI used where co-transduction of vectors encoding p19Arf and IFNβ was performed, the MOI of the GFP condition was equal to the total MOI of the co-transduction. Where p19Arf or IFNβ viruses were to be used separately, the total MOI was completed with the GFP vector. In this way, MOIs and gene dosages were always equal across all conditions.

### Detection of GFP by flow cytometry

After 24 h of incubation post-transduction, cells were trypsinized, washed with 1× PBS, and resuspended in 1 mL of 1× PBS before observation (Attune, Thermo Fisher), and analyzed using the Attune Cytometric Software.

### ELISA for the quantification of mouse IFN&mac_beta;

MSCs were transduced as described above and supernatant was collected 48 h post-transduction and stored at -80°C. ELISA was performed using the mouse IFNβ kit from PBL Biomedical (USA) and absorbance at 450 nm was analyzed using a Victor plate reader (Perkin-Elmer, USA).

### MTT assay

MSCs were plated in 96-well plates, 1×10^3^ cells/well, and the next day transduced with the adenoviral vectors. The plates were then incubated for 24, 48, or 72 h before adding 5 mg/mL MTT in PBS, incubating at 37°C for 4 h then solubilizing the converted substrate using 20% SDS/50% DMF, pH 4.7. The next day, absorbance at 570 nm was examined using a Victor plate reader (Perkin-Elmer). Each experimental condition was performed in quadruplicate and the biological assays were performed on three independent occasions.

### Co-culture assays

MSCs were plated in 6-well dishes and transduced during 6 h before washing three times with 1× PBS. Next, the MSCs were trypsinized and counted. In parallel, the B16-GFP cells were also transduced as indicated, incubated at 37°C for 6 h, washed, collected, and counted. Then, equal proportions of each cell line (1×10^4^ cells/line) were seeded together in 6-well dishes in DMEM/F12 and 20% FBS. Ten random fields were photographed (EVOS FL, Thermo Fisher) at the indicated time points and the fluorescent (B16-GFP) or non-fluorescent (MSC) populations were manually counted with the assistance of ImageJ software (National Institutes of Health, USA).

### Statistical analysis

Statistical analysis was performed comparing individual experimental conditions with the control and using Student's *t*-test; P<0.05 was considered significant. Data are reported as the average and standard error of measurement. Biological and technical replicates are given in each figure legend. Analyses were performed using GraphPad Prism 5.0 (USA) and Microsoft Excel (USA).

## Results

Mouse MSCs were isolated from adipose tissue and cultivated for three or eight passages before testing cell surface markers. As expected, the MSCs displayed stem cell markers Sca-1 and CD29, but did not present lineage-specific markers CD11b, CD31, or CD45 at passage 3 (Supplementary Figure S1). However, this pattern did not persist at passage eight when Sca-1 staining was reduced (Supplementary Figure S1). Thus, care was taken to use low passage numbers (3 to 5) for all experiments. The low-passage MSCs were permissive for differentiation into adipocytes, chondrocytes, and osteocytes (Supplementary Figure S1), confirming that the cell population isolated consisted principally of MSCs.

**Figure 1:**
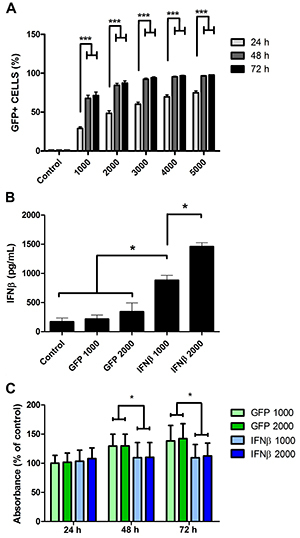
Transduction of mesenchymal stem cells (MSCs) with RGD-modified adenoviral vectors. **A**, MSCs were either not transduced (Control) or transduced with increasing multiplicity of infection (1000 to 5000) using AdRGD-PG-eGFP and the percentage of green fluorescent protein positive cells (GFP+) was determined by flow cytometry. **B**, MSCs were transduced using AdRGD-PG-eGFP (GFP) or AdRGD-PG-IFNβ (IFNβ) and, 48 h later, accumulation of IFNβ in the supernatant was examined by ELISA. **C**, Metabolic activity of MSCs was determined by MTT assay 24, 48, or 72 h post-transduction. Data are reported as the average and standard error of 6 (**A**) or 3 (**B** and **C**) independent assays. *P<0.05, ***P<0.001, statistical analysis was performed comparing individual experimental conditions with the corresponding control using the Student's *t*-test.

Next, the MSCs were transduced with the AdRGD-PG-eGFP adenoviral vector (non-replicating, serotype 5, RGD fiber modification, PGTxβ chimeric p53 responsive promoter) and evaluated by flow cytometry. As seen in Figure 1A, transduction efficiency increased in proportion to the MOI used. Since MOIs of 1000 and 2000 did not induce any apparent toxicity, these MOIs were tested for the production of IFNβ upon transduction with the AdRGD-PG-IFNβ virus (Figure 1B). The impact of IFNβ production on MSC metabolism (viability) was tested using the MTT assay, revealing statistically significant, but limited, reduction in cellular activity (Figure 1C). In contrast, MSCs were sensitive to p19Arf gene transfer (Supplementary Figure S2). These assays showed that MSCs were susceptible to AdRGD transduction, expressed the transgene through the p53-responsive promoter, and were minimally affected by IFNβ production.

**Figure 2:**
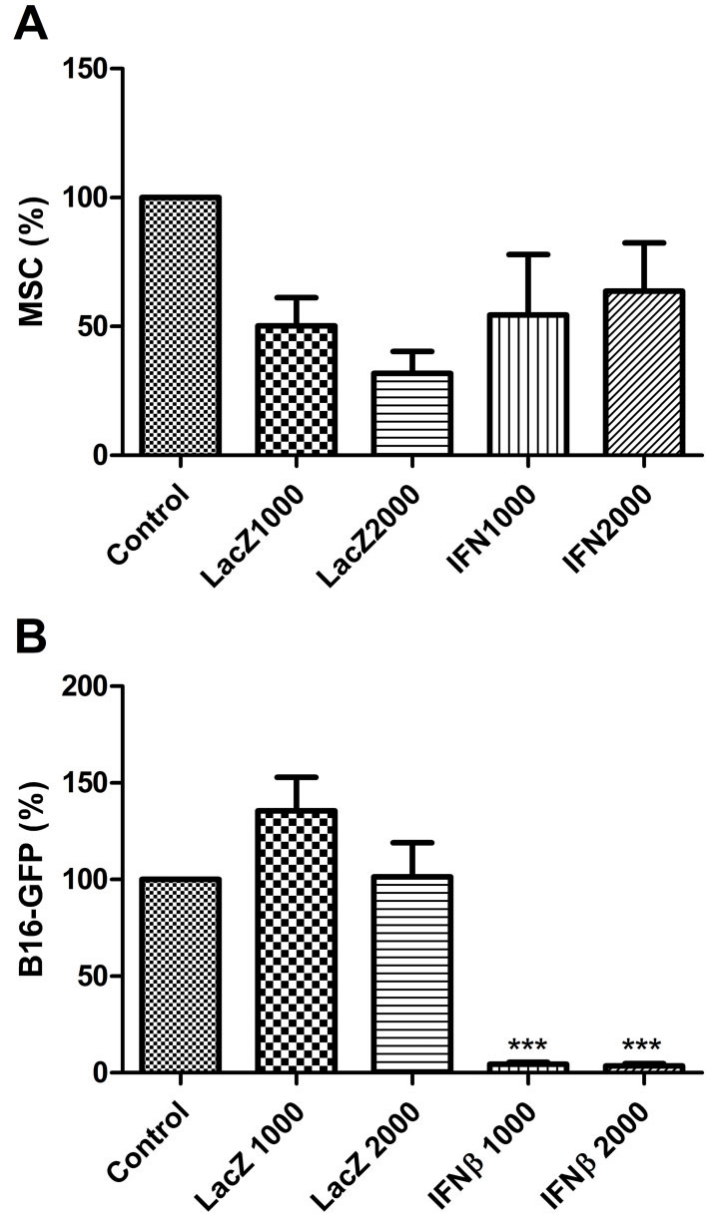
Mesenchymal stem cells (MSCs) producing interferon-β (IFNβ) inhibited co-cultured B16 mouse melanoma cells. MSCs were either not transduced (Control) or transduced with AdRGD-CMV-LacZ (LacZ) or AdRGD-PG-IFNβ (IFNβ) at the indicated multiplicity of infection (1000 or 2000) and then co-cultured with B16 cells with stable expression of eGFP (B16-GFP) and, 96 h later, each cell population was counted based on the presence or absence of eGFP expression. Quantification of the (**A**) MSC or (**B**) B16-GFP populations is reported as the average and standard error from three independent experiments, normalized by the value obtained for the Control group. ***P<0.001, statistical analysis was performed comparing individual experimental conditions with the control using Student's *t*-test. GFP: green fluorescent protein.

To model MSC-mediated payload delivery, we transduced MSCs with the IFNβ or LacZ adenoviral vector, AdRGD-CMV-LacZ, then co-cultured these cells along with B16-GFP cells (mouse melanoma with stable expression of eGFP). After 96 h of incubation at 37°C, the cells were photographed (as shown in Supplementary Figure S3) and each population (eGFP-negative or positive, indicating MSC or B16, respectively) was counted. We observed that the population of MSCs was not significantly reduced by their transduction with either LacZ or IFNβ (Figure 2A), yet the co-cultured B16-GFP population was significantly depleted, though this effect required a relatively long incubation period of 96 h (Figure 2B).

**Figure 3:**
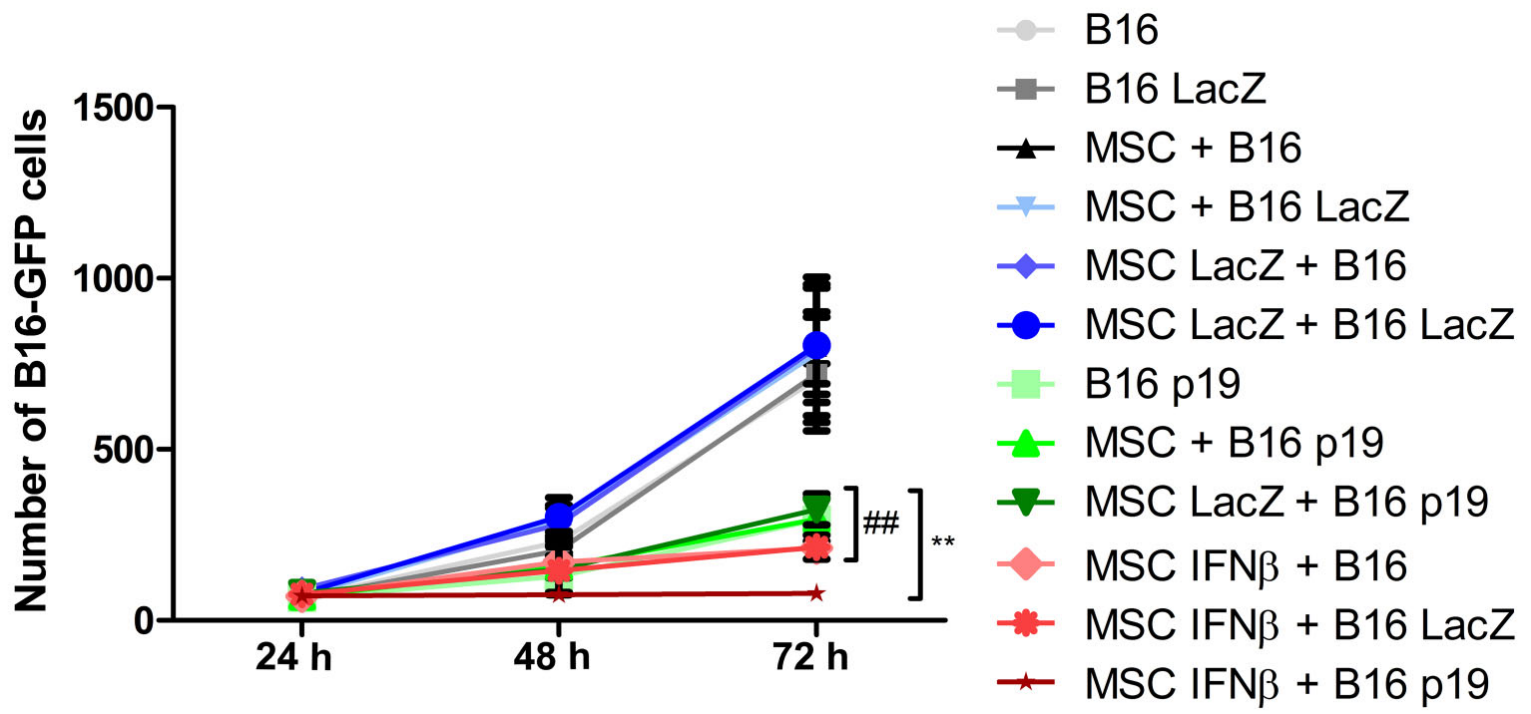
p19Arf sensitizes B16 cells to interferon-β (IFNβ) produced by mesenchymal stem cells (MSCs). MSCs were either not transduced (MSC) or transduced with AdRGD-CMV-LacZ (MSC LacZ) or AdRGD-PG-IFNβ (MSC IFNβ) and then co-cultured with B16 cells with stable expression of eGFP (B16-GFP) that were also either not transduced (B16) or transduced with either AdRGD-CMV-LacZ (B16 LacZ) or AdRGD-PG-p19 (B16 p19). At the indicated time points, cells were photographed and the B16 cell population was counted based on presence of eGFP expression. Data are reported as the average and standard error from three independent experiments. **P<0.01 comparing individual experimental conditions with the control (MSC LacZ + B16 LacZ, 72 h) using the Student's *t*-test. ^##^P<0.01 comparing individual experimental conditions with MSC IFNβ + B16 p19 (72 h) using Student's *t*-test.

We investigated whether the presence of p19Arf in the B16 melanoma cells would sensitize them to the effects of IFNβ delivered by the MSCs. In this co-culture assay, the transduction of melanoma cells with AdRGD-PG-p19Arf yielded essentially complete inhibition in the presence of IFNβ delivered by MSCs (Figure 3). In comparison, the addition of p19Arf to the tumor cells was insufficient for the blockage of their proliferation when MSCs did not carry IFNβ. Again, IFNβ delivered by MSCs did lead to the inhibition of the melanoma cells, but this was more effective in the presence of p19Arf. The presence or absence of the control virus encoding LacZ did not influence the results, indicating that non-specific responses did not play a part in the effect of p19Arf and IFNβ on the cell populations. These data show that p19Arf sensitizes B16 melanoma cells to IFNβ delivered by MSCs.

## Discussion

Here, we have shown that MSCs derived from mouse adipose tissue can be modified with our adenoviral vectors and used to deliver a therapeutic payload, IFNβ. This cytokine did not cause extensive killing of the MSCs, yet their production of IFNβ did inhibit B16 mouse melanoma cells in a co-culture assay. Strikingly, the presence of p19Arf in the B16 cells further sensitized them to the effects of IFNβ delivered by the MSCs. While this work represents a critical first step and much work remains to be done, we concluded that MSCs can be employed as an interesting component of our combined p19Arf + IFNβ gene transfer approach.

We propose that use of MSCs as a carrier of IFNβ may provide advantages over intratumoral gene therapy. In particular, the MSC approach is expected to be transient when applied *in vivo*, since cells may migrate out of the tumor, differentiate, or die (9,10). The adenovirus that they carry cannot replicate and is not integrated in the host genome, therefore this approach is incompatible with stable, long term exogenous gene expression (23,24). The transient nature of the technology should be sufficient to bring about cell killing and stimulation of an anti-tumor immune response. Though this point remains to be shown experimentally in our model, such responses have been reported (25–29). In addition, unresolved type I interferon signaling may actually be detrimental since it may induce immunosuppression (1), thus limiting the exposure to IFNβ may be beneficial.

The inclusion of p19Arf in our gene transfer approach is intended to bring about cell death, an outcome that would resolve the issues related to prolonged exposure to IFNβ and which would provide both antigens and signaling that should contribute to an anti-tumor immune response. In our previous studies, we have noted that either IFNβ or p19Arf alone can indeed kill tumor cells, yet their combination enhances this effect (12–14,30). In addition, combined, but not individual, gene transfer was associated with the induction of immunogenic cell death (12,13). However, in those assays, p19Arf and IFNβ were produced by the same cell.

Since IFNβ is a secreted protein, it need not be produced by the target cell and may exert an effect when provided in a paracrine manner. In a previous study, we have noted that tumor cells producing IFNβ can kill co-cultured, naive tumor cells and that this effect is enhanced in the presence of p19Arf (20). In a recent study, we have shown that transduction of endothelial cells with our AdRGD-PG-IFNβ does not kill these cells, yet the secretome that they produce does induce death in B16 cells (20), a situation that is quite similar to the one exposed here. However, when the tumor cells produce IFNβ, the endothelial cells are killed by either co-culture or conditioned medium. Moreover, treatment of endothelial cells with recombinant IFNβ protein does not induce cell death, implying that IFNβ is not sufficient for killing the endothelial cells (20). While this phenomenon has yet to be studied in the MSC model, the possibility exists that IFNβ is acting in concert with additional factors produced by the transduced cells.

Clearly, additional work is required to reveal the molecular mechanisms that contribute to tumor cell killing in response to IFNβ delivered by MSCs and *in vivo* experiments would shed light on MSC homing to the tumor and its consequences on tumor progression. We propose that, upon treatment, the dynamics within the tumor microenvironment may reveal beneficial anti-tumor responses, including immune activation, inhibition of angiogenesis, and induction of cell death. The assays described here represent a point of departure for future studies that may reveal the interplay between gene transfer, cell therapy, and therapeutic transgene activity.

## Supplementary Material

Click here to view [pdf].
